# Allergen extract-based sensitization patterns in mono-sensitized versus multi-sensitized patients with allergic rhinitis in Ningxia, China

**DOI:** 10.3389/falgy.2026.1848987

**Published:** 2026-08-31

**Authors:** Yuqiao Zhang, Peng Liu, Fengxia Yang, Xiaohui Yan, Xueliang Shen, Xiyuan Yan, Ruixia Ma

**Affiliations:** 1 The Second Clinical Medical College of Ningxia Medical University, Yinchuan, China; 2 Otorhinolaryngology Head and Neck Surgery Hospital, The First People’s Hospital of Yinchuan, Yinchuan, China; 3 Department of Otorhinolaryngology Head and Neck Surgery, General Hospital of Ningxia Medical University, Yinchuan, China

**Keywords:** allergens, cross-sensitization, multi-sensitization, rhinitis, allergic, sIgE detection

## Abstract

**Background:**

Patients with allergic rhinitis (AR) and multi-sensitization experience more severe symptoms and a higher risk of asthma complications compared to those with mono-sensitization. Allergen proportion is closely linked to geographical distribution, yet detailed profiles of multi-sensitized AR patients in Ningxia, China, remain underexplored.

**Objective:**

To investigate the distribution of inhaled allergens and associated factors in multi-sensitized AR patients in Ningxia, providing a data foundation for individualized treatment.

**Methods:**

A retrospective analysis included 1,180 AR patients diagnosed at Yinchuan First People's Hospital (January 2018–August 2022) who underwent serum allergen-specific IgE (sIgE) testing. After excluding 70 patients who tested negative for all 10 inhalant allergens, 1,110 patients with at least one positive inhalant allergen were analyzed. Among them, 826 patients with ≥2 positive inhalant allergens (multi-sensitized group) and 284 with a single positive inhalant allergen (mono-sensitized group) were compared.

**Results:**

(1) 74.4% (826/1,110) of sensitized AR patients had multi-sensitization. (2) The predominant inhaled allergens were mugwort (74.1%), ragweed (54.7%), and tree mix (49.2%). (3) The top allergens by mean sIgE intensity were mugwort (3.65), cat dander (3.06), and house dust (3.00). (4) Multi-sensitization was significantly associated with age (*χ*^2^ = 116.032, *P* < 0.001), food allergy comorbidity (*χ*^2^ = 107.622, *P* < 0.001), season of consultation (*χ*^2^ = 58.475, *P* < 0.001), COVID-19 pandemic period (*χ*^2^ = 85.513, *P* < 0.001), and ethnicity (*χ*^2^ = 64.627, *P* < 0.001). Post-COVID-19, the proportion of multi-sensitization decreased from 80.0% to 55.2%.

**Conclusions:**

Pollen allergens, particularly mugwort, are the most common triggers for multi-sensitized AR patients in Ningxia, demonstrating distinct regional and demographic patterns. Comprehensive allergen testing, local pollen monitoring, and exposure reduction are crucial for managing this high-risk subgroup.

## 1 Introduction

Allergic rhinitis (AR) is an immunoglobulin E (IgE)-mediated inflammatory disorder of the nasal mucosa, characterized by sneezing, rhinorrhea, nasal itching, and congestion. Its global and national proportion continues to rise annually (1–3), presenting a significant public health burden. In the Ningxia Region, one study reported an AR proportion of 13.06% among residents aged 5–70 years (4). Allergens, particularly inhalant allergens such as pollens, dust mites, and animal dander, are the primary triggers of AR. Their distribution varies considerably with geographic location and climate, influencing local sensitization patterns (5).

Among AR patients, those with multi-sensitization (allergy to two or more allergens) face a more severe clinical profile compared to their single-sensitization counterparts (6). The number of sensitizing allergens themselves is a recognized risk factor influencing disease onset, progression, and outcomes (7). Inhaled allergens, as the primary sensitizers that directly interact with the nasal mucosa, are of paramount importance in AR pathogenesis. Avoiding known allergens is a cornerstone of AR management, making accurate allergen identification through testing a critical component of prevention and control strategies. The distribution of allergens and patterns of multi-sensitization are not uniform but are profoundly shaped by regional geographical and climatic factors (8).

While the general allergen profile in Ningxia has been described (9), a detailed analysis focusing specifically on the characteristics of multi-sensitization, a state associated with greater disease severity, remains lacking.

**Figure 1:**
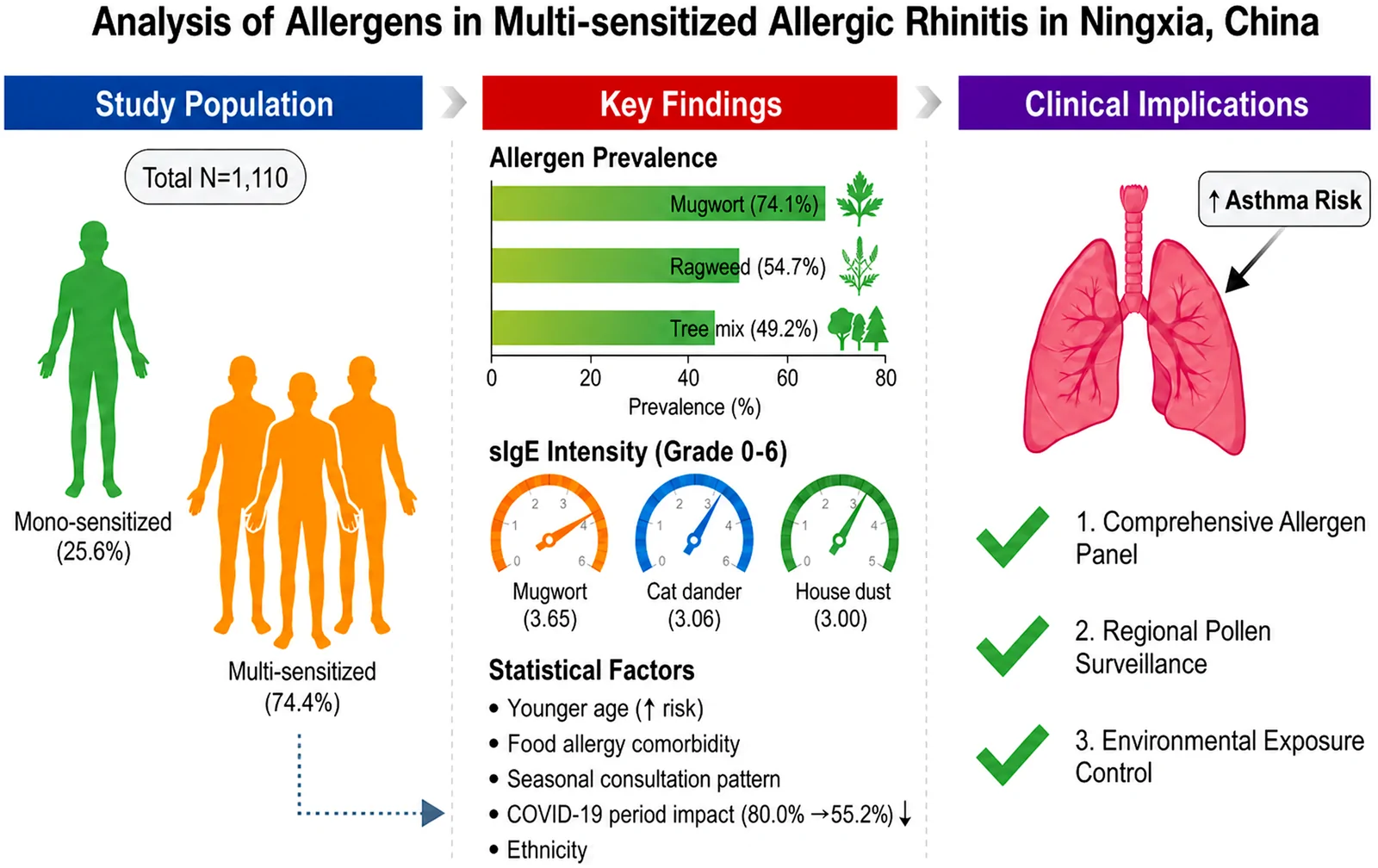
Summary of multisensitization patterns, associated factors, and clinical implications in 1,110 AR patients from Ningxia, China.

This study focused exclusively on patients with multiple inhalant allergen sensitization in Ningxia, China. We seek to delineate the distribution patterns, predominant allergen combinations, and associated factors (e.g., age, sex, co-sensitization to food allergens, season) in this clinically significant subgroup. The findings are intended to provide a data-driven foundation for developing more precise and individualized management strategies for multi-sensitized AR patients in this region. A visual summary of the study design, key results, and clinical implications is provided (Figure 1).

## 2 Materials and methods

### 2.1 Clinical data

This retrospective study initially included all consecutive patients diagnosed with allergic rhinitis at the outpatient clinic of the Otorhinolaryngology Head and Neck Surgery Hospital of Yinchuan First People's Hospital between January 2018 and August 2022. A total of 1,180 patients were included, and all of them underwent serum specific immunoglobulin E (sIgE) testing. After excluding 70 patients who tested negative for all 10 inhalant allergens, 1,110 patients with positive results for at least one inhalant allergen were included in the final analysis. Among these, 284 patients had exactly one positive inhalant allergen (mono-sensitized group), and 826 patients had two or more positive inhalant allergens (multi-sensitized group). All consecutive patients meeting the inclusion criteria during the study period were enrolled; no formal sample size calculation was performed, as this was a retrospective descriptive study.

A portion of the patient data in this study was derived from the same source as a previously published article in Chinese (9). This study extended the enrollment period of a previously published Chinese study (which covered January 2018–December 2021) to August 2022, thereby expanding the original database. The current manuscript presents a novel and distinct analysis, focusing specifically on comparing multi-sensitized versus mono-sensitized patients and exploring factors associated with multi-sensitization. The research questions, analytical methods, and conclusions are unique to this work.

### 2.2 Inclusion and exclusion criteria

#### 2.2.1 Inclusion criteria

(1) Permanent residents of the Ningxia Region; (2) Diagnosis of AR based on the Chinese Guideline for Diagnosis and Treatment of Allergic Rhinitis (2022, revision) (10), presenting with two or more symptoms such as nasal itching, sneezing, watery mucus, nasal obstruction, and decreased sense of smell, and symptoms persist or accumulate for more than 1 h every day and lasting for at least 4 weeks; Signs: gray nasal mucosa, gray edema, nasal watery secretions (10). (3) Serum sIgE test results indicating sensitization to ≥2 types of inhalant allergens (for the multi-sensitized group) or to a single type (for the mono-sensitized group); (4) No history of mental illness or substance abuse, with normal cognitive function.

#### 2.2.2 Exclusion criteria

(1) Comorbid with other nasal diseases (e.g., chronic rhinosinusitis with nasal polyps, non-allergic rhinitis); (2) Presence of severe systemic immune diseases or malignancies; (3) Use of systemic corticosteroids or immunosuppressants within 4 weeks prior to testing; (4) History of allergen immunotherapy or treatment with omalizumab (anti-IgE) within the past 6 months; (5) Incomplete clinical data or test results (4); (6) Confirmed acute COVID-19 infection at the time of presentation (due to overlapping symptoms such as anosmia and sneezing).

The study protocol was approved by the Ethics Committee of the Second Affiliated Hospital of Ningxia Medical University (Yinchuan First People's Hospital) (Approval No: 2023-028). The requirement for informed consent was waived due to the retrospective nature of the study.

### 2.3 Detection method

Extract-based sIgE testing remains the most practical and cost-effective screening tool for large-scale epidemiological studies in routine clinical practice. While component-resolved diagnostics offer higher molecular resolution, they are not routinely available in many clinical settings due to their substantially higher cost. Therefore, this study was designed to characterize the real-world sensitization patterns using the diagnostic platform commonly accessible to clinicians in this region. The routine diagnostic panel used in this study was determined by the hospital based on the most prevalent sensitizers identified in the local population and the clinical affordability within this region, reflecting the actual diagnostic capacity in routine practice.

Specific IgE antibodies were measured using the ImmunoCAP® system (Phadia AB, Sweden), a quantitative fluorescence enzyme immunoassay. The sensitivity and specificity of this method are ISO 15189 certified, enabling accurate detection of IgE antibody levels against specific allergens in serum. This method has reported sensitivity and specificity >85% for common aeroallergens (10, 11).

The assay panel included 10 common inhalant allergens: The assay panel included 10 common inhalant allergens: Tree Mix (poplar/willow/elm) (tx5), Ragweed (w1), Mugwort (w6), House Dust Mite Mix (Dermatophagoides pteronyssinus/D. farinae) (dx1), House dust (h1), Cat Dander (e1), Dog Dander (e2), Cockroach (German) (i6), Mold Mix (Penicillium notatum/Cladosporium herbarum/Aspergillus fumigatus/Alternaria alternata) (mx1), and Humulus scandens (w22). Additionally, 11 food allergens were tested: Egg White (f1), Cow's Milk (f2), Peanut (f13), Soybean (f14), Beef (f27), Mutton (f88), Marine Fish Mix (fx2), Shrimp (f24), Crab (f23), Fruit Mix (fx3), and Nut Mix (fx1). It should be noted that the panel did not include grass pollen allergens (e.g., timothy, ryegrass, Bermuda grass), as these are less prevalent sensitizers in the Ningxia region based on prior epidemiological data (4, 12).

The present study focuses on sensitization to inhalant allergens. Therefore, the terms “multi-sensitization” and “number of allergens” hereafter specifically refer to sensitization to two or more types of inhalant allergens. Venous blood was drawn from all patients, blood samples were collected in plain vacuum tubes, allowed to clot at room temperature for 30 min, and centrifuged at 3000 rpm for 10 min. Serum aliquots were stored at −20 °C and analyzed within 7 days of collection, consistent with the manufacturer's stability guidelines. Skin prick testing (SPT) is a cheaper alternative for allergen detection but was not available in this setting.

### 2.4 Result determination and strength calculation

Results were interpreted and graded (0−6) according to the Chinese Guidelines for the Diagnosis and Treatment of Allergic Rhinitis (10) (2022, revision), with a concentration ≥0.35 U/mL (Grade ≥1) defined as positive. For multi-sensitized patients, the “total allergen intensity” was defined as the sum of the grades of all positive allergens, and the “individual average intensity” was calculated as the total intensity divided by the number of positive allergen types. These indices are exploratory descriptive measures created for internal comparison within this study; they have not been clinically validated and should be interpreted with caution.

### 2.5 Grouping

Patients were categorized into the following groups for analysis: (1) by sensitization status: multi-sensitized (≥2 positive inhalant allergens) vs. mono-sensitized (1 positive inhalant allergen); (2) by age: 0−4 years of age (infant group), 5−11 years (child group), 12−18 years of age (adolescent group), 19−35 years of age (young group), 36−59 years of age (middle age group), and 60 years of age and older (elderly group); (3) by season of consultation: spring (March–May), summer (June–August), autumn (September–November), and winter (December–February); (4) by period relative to the COVID-19 pandemic: pre-pandemic (January 2018–December 2019) and post-pandemic (January 2020–August 2022).

### 2.6 Method of statistics

**Figure 2:**
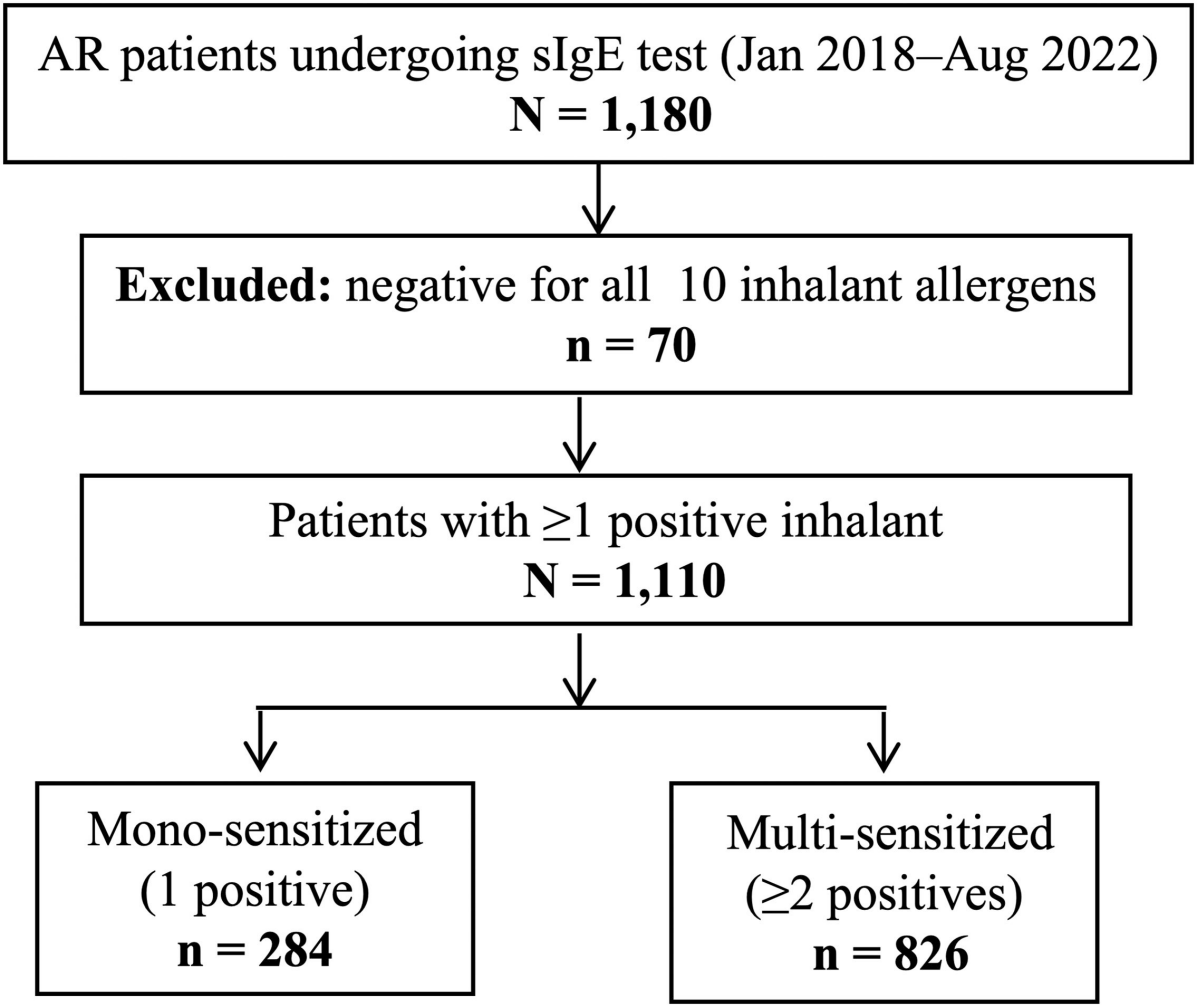
Patient screening flowchart.

Statistical analysis was performed using SPSS software (version 26.0). Categorical data are presented as numbers (percentages) and were compared using the chi-square test. A *P*-value of <0.05 was considered statistically significant. Given the exploratory and descriptive nature of this study, we used univariate analyses to identify associations. Multivariate logistic regression was not performed due to the relatively small sample sizes in certain subgroups (e.g., ethnic minorities, older adults) and to avoid overfitting and unstable estimates. A flow diagram detailing patient selection is presented in Figure 2.

## 3 Results

### 3.1 General situation

A total of 1,110 AR patients with positive inhalant allergen tests were included in the final analysis. Among them, 284 (25.6%) were mono-sensitized and 826 (74.4%) were multi-sensitized. Comparative analysis between mono-sensitized and multi-sensitized patients revealed no significant difference in gender distribution (*P* = 0.587). However, significant differences were observed in age distribution (*P* < 0.01), season of consultation (*P* < 0.001), co-sensitization to food allergens (*P* < 0.001), and ethnicity (*P* < 0.001). The detailed distribution is presented in Table 1. The significant differences observed in Table 1 (e.g., higher proportion of young adults and food-allergic patients in the multi-sensitized group) likely reflect greater cumulative allergen exposure and a more active immune response in these individuals, though causality cannot be inferred from this cross-sectional design.

**Table 1:** Comparison of baseline characteristics between mono-sensitized and multi-sensitized AR patients.

Characteristic	Total positive patients (*n* = 1110)	Mono-sensitized group (*n* = 284)	Multi-sensitized group (*n* = 826)	Proportion of multi-sensitization (%)	*χ* ^2^	*P* value
Sex						
Male	500	124	376	75.2	0.295	0.587
Female	610	160	450	73.8		
Age (years)						
0∼4	8	2	6	75.0	16.760	0.005*
5∼11	150	34	116	77.3		
12∼18	104	30	74	71.2		
19∼35	310	96	214	69.0		
35∼59	442	89	353	79.9		
≥60	96	33	63	65.6		
Season of Consultation						
Spring (Mar–May)	36	22	14	38.9	37.638	< 0.001**
Summer (Jun–Aug)	932	210	722	77.5		
Autumn (Sep–Nov)	106	38	68	64.2		
Winter (Dec–Feb)	36	14	22	61.1		
The COVID-19 pandemic Period						
Pre-pandemic (2018–2019)	860	172	688	80.00	62.570	< 0.001**
Post-pandemic (2020–2021)	250	112	138	55.2		
Co-sensitization to Food Allergens						
Yes	266	30	236	88.7	37.612	< 0.001**
No	844	254	590	69.9		
Ethnic						
Han	626	123	503	80.4	64.627	< 0.001**
Hui	226	49	177	78.3		
Manchu	8	0	8	100.0		
Unknown	250	112	138	55.2		

Comparison of baseline characteristics between mono-sensitized and multi-sensitized AR patients. Categorical data are presented as number (*n*) and percentage (%). Group comparisons were performed using the chi-square (*χ*^2^) test. A *P* value of < 0.05 was considered statistically significant.

Percentages may not sum to 100% due to rounding.

* *P* < 0.01.

** *P* < 0.001.

### 3.2 Distribution of number and species of sensitization

Among multi-sensitized patients, the most prevalent inhalant allergens were mugwort (74.1%), ragweed (54.7%), and tree mix (49.2%). The number of positive inhalant allergens per patient ranged from 2 to 10 (mean ± SD: 3.81 ± 1.7). Among patients sensitized to exactly two allergens, the combination of mugwort and ragweed was the most frequent (66 cases, 28.9% of double-sensitized patients). For those sensitized to three allergens, the combination of treemix, mugwort, and ragweed was predominant (88 cases, 44.0% of triple-sensitized patients), representing 44.00% of the total number of triple allergens (Tables 2, 3). A clear inverse relationship was observed: the number of patients declined progressively as the number of sensitizing allergen types increased (Table 4).

**Table 2:** Prevalence and mean positive intensity of major inhalant allergens in multi-sensitized patients (*n* = 826).

Allergen	Positive cases, *n*	Proportion, %	Mean positive intensity (grade)
Mugwort	612	(74.1)	3.65
Ragweed	452	(54.7)	2.98
Tree Mix (Poplar/Willow/Elm)	406	(49.2)	2.93
Cockroach (German)	196	(23.7)	2.51
Mold Mix (*P. notatum/C. herbarum/A. fumigatus/A. alternata*)	122	(14.8)	2.30
Humulus scandens	124	(15.0)	2.55
House Dust Mite Mix (*D. pteronyssinus/D. farinae*)	118	(14.3)	2.39
Cat Dander	98	(11.9)	3.06
Dog Dander	20	(2.4)	2.20
House dust	22	(2.7)	3.00

Categorical data are presented as number (*n*) and percentage (%).

**Table 3:** Prevalence of specific allergens among multi-sensitized patients, stratified by number of sensitizations [*n* (%)].

Allergen	Number of sensitization [*n* (%)]									*χ* ^2^	*P* value
	2 (*n* = 228)	3 (*n* = 200)	4 (*n* = 136)	5 (*n* = 130)	6 (*n* = 68)	7 (*n* = 32)	8 (*n* = 24)	9 (*n* = 6)	10 (*n* = 2)		
Mugwort	148 (64.9)	166 (83.0)	124 (91.2)	128 (98.5)	60 (88.2)	30 (93.8)	22 (91.7)	6 (100.0)	2 (100.0)	276.832	< 0.001*
Ragweed	86 (37.7)	132 (66.0)	116 (85.3)	110 (84.6)	58 (85.3)	30 (93.8)	22 (91.7)	6 (100.0)	2 (100.0)	469.862	< 0.001*
Tree Mix	64 (28.1)	132 (66.0)	120 (88.2)	114 (87.7)	64 (94.1)	30 (93.8)	24 (100.0)	6 (100.0)	2 (100.0)	529.426	< 0.001*
Cockroach	48 (21.1)	42 (21.0)	42 (30.9)	70 (53.8)	36 (52.9)	30 (93.8)	20 (83.3)	4 (66.7)	2 (100.0)	205.544	< 0.001*
Mold Mix	32 (14.0)	44 (22.0)	26 (19.1)	72 (55.4)	40 (58.8)	28 (87.5)	22 (91.7)	6 (100.0)	2 (100.0)	263.028	< 0.001*
Humulus scandens	6 (2.6)	26 (13.0)	70 (51.5)	60 (46.2)	48 (70.6)	20 (62.5)	10 (41.7)	6 (100.0)	2 (100.0)	364.695	< 0.001*
Dust Mite Mix	28 (12.3)	20 (10.0)	24 (17.6)	40 (30.8)	50 (73.5)	24 (75.0)	18 (75.0)	6 (100.0)	2 (100.0)	320.532	< 0.001*
Cat Dander	24 (10.5)	26 (13.0)	14 (10.3)	36 (27.7)	26 (38.2)	10 (31.3)	22 (91.7)	6 (100.0)	2 (100.0)	226.614	< 0.001*
Dog Dander	12 (5.3)	6 (3.0)	2 (1.5)	10 (7.7)	8 (11.8)	14 (43.8)	18 (75.0)	6 (100.0)	2 (100.0)	277.868	< 0.001*
House dust	8 (3.5)	6 (3.0)	6 (4.4)	10 (7.7)	10 (14.7)	6 (18.8)	14 (58.3)	6 (100.0)	2 (100.0)	268.364	< 0.001*

Categorical data are presented as number (*n*) and percentage (%). Group comparisons were performed using the chi-square (*χ*^2^) test. A *P* value of <0.05 was considered statistically significant.

Percentages may not sum to 100% due to rounding.

* *P* < 0.001.

**Table 4:** Allergen intensity scores in multi-sensitized patients stratified by number of sensitizations.

Number of sensitizations	Number of sensitization (*n*)								
	2 (*n* = 228)	3 (*n* = 200)	4 (*n* = 136)	5 (*n* = 130)	6 (*n* = 68)	7 (*n* = 32)	8 (*n* = 24)	9 (*n* = 6)	10 (*n* = 2)
Ratio of Patients (%)	27.6	24.2	16.5	15.7	8.2	3.9	2.9	0.7	0.2
Total Intensity Score (Mean)	4.32	7.30	9.96	12.79	13.41	16.56	17.42	19.00	23.00
Individual Average Intensity (Mean)	2.29	2.34	2.57	2.55	2.24	2.37	2.18	2.11	2.30

Total intensity score is the sum of grades for all positive allergens. Individual average intensity is the total score divided by the number of positive allergen types. These are descriptive indices generated for internal comparison and have not been clinically validated. Categorical data are presented as number (*n*) and percentage (%).

### 3.3 Number and intensity distribution of sensitization

The allergens with the highest mean individual intensity scores among multi-sensitized patients were mugwort (3.65), cat dander (3.06), and house dust (3.00). The total allergen intensity score increased with the number of sensitizations, ranging from 4.32 for double sensitization to 23.00 for deca-sensitization. Conversely, the individual average intensity score remained relatively stable across different sensitization number groups, with an overall mean of 2.38 ± 0.81 (Table 4). These findings suggest that while total sIgE burden increases with the number of sensitizations, the average intensity per allergen remains relatively constant.

### 3.4 Distribution of the number of oversensitization in different age and sex groups

Analysis of sensitization number by age and sex revealed significant patterns. A significant difference in the number of sensitizations was observed across different age groups (*P* < 0.001). Significant differences in sensitization number between sexes were found specifically within the 19–35 years age group (*P* = 0.004), and in the overall cohort (*P* = 0.002), with females generally showing higher sensitization counts. The detailed distribution is presented in Table 5.

**Table 5:** Distribution of the number of sensitizations stratified by age and sex in multi-sensitized patients [*n* (%)].

Age (years)	Sex	Number of sensitization [*n* (%)]									Row total (*n*)	*χ* ^2^	*P* value
		2	3	4	5	6	7	8	9	10			
0∼4	Male	2 (50.0)	2 (50.0)	–	–	–	–	–	–	–	4	2.667	0.264
	Female	2 (100)	–	–	–	–	–	–	–	–	2		
	Subtotal	4 (66.7)	2 (33.3)	–	–	–	–	–	–	–	6	116.032	< 0.001**
5∼11	Male	16 (21.6)	14 (18.9)	24 (32.4)	18 (24.3)	–	–	2 (2.7)	–	–	74	9.469	0.149
	Female	12 (28.6)	12 (28.6)	8 (19.0)	8 (19.0)	–	2 (4.8)	–	–	–	42		
	Subtotal	28 (24.1)	26 (22.4)	32 (27.6)	26 (22.4)	–	2 (1.7)	2 (1.7)	–	–	116		
12∼18	Male	8 (20.0)	6 (15.0)	12 (30.0)	8 (20.0)	6 (15.0)	–	–	–	–	40	10.286	0.113
	Female	8 (23.5)	8 (23.5)	8 (23.5)	2 (5.9)	4 (11.8)	4 (11.8)	–	–	–	34		
	Subtotal	16 (21.6)	14 (18.9)	20 (27.0)	10 (13.5)	10 (13.5)	4 (5.4)	–	–	–	74		
19∼35	Male	10 (13.2)	16 (21.1)	22 (28.9)	10 (13.2)	10 (13.2)	6 (7.9)	–	2 (2.6)	–	76	22.493	0.004*
	Female	30 (21.7)	42 (30.4)	12 (8.7)	24 (17.4)	10 (7.2)	10 (7.2)	6 (4.3)	4 (2.9)	–	138		
	Subtotal	40 (18.7)	58 (27.1)	34 (15.9)	34 (15.9)	20 (9.3)	16 (7.5)	6 (2.8)	6 (2.8)	–	214		
35∼59	Male	46 (31.5)	32 (21.9)	24 (16.4)	26 (17.8)	10 (6.8)	–	8 (5.5)	–	–	146	12.412	0.134
	Female	69 (33.3)	50 (24.2)	22 (10.6)	30 (14.5)	22 (10.6)	6 (2.9)	6 (2.9)	–	2 (1.0)	207		
	Subtotal	115 (32.6)	82 (23.2)	46 (13.0)	56 (15.9)	32 (9.1)	6 (1.7)	14 (4.0)	–	2 (0.6)	353		
≥60	Male	14 (38.9)	12 (33.3)	2 (5.6)	2 (5.6)	4 (11.1)	2 (5.6)	–	–	–	36	9.999	0.189
	Female	11 (40.7)	6 (22.2)	2 (7.4)	2 (7.4)	2 (7.4)	2 (7.4)	2 (7.4)	–	–	27		
	Subtotal	25 (39.7)	18 (28.6)	4 (6.3)	4 (6.3)	6 (9.5)	4 (6.3)	2 (3.2)	–	–	63		
Overall	Male	96 (25.5)	82 (21.8)	84 (22.3)	64 (17.0)	30 (8.0)	8 (2.1)	10 (2.7)	2 (0.5)	–	376	25.916	0.002*
	Female	132 (29.3)	118 (26.2)	52 (11.6)	66 (14.7)	38 (8.4)	24 (5.3)	14 (3.1)	4 (0.9)	2 (0.4)	450		
	Grand Total	228 (27.6)	200 (24.2)	136 (16.5)	130 (15.7)	68 (8.2)	32 (3.9)	24 (2.9)	6 (0.7)	2 (0.2)	826		

Data are presented as *n* (row %). Percentages represent the distribution of sensitization numbers within each age-sex subgroup.

– indicates zero cases. Subtotal and Grand Total percentages are calculated relative to their respective row totals.

Group comparisons were performed using the chi-square (χ^2^) test. A *P* value of <0.05 was considered statistically significant. Percentages may not sum to 100% due to rounding.

* *P* < 0.01.

** *P* < 0.001.

### 3.5 Distribution of the number of allergens in different food sensitization groups

Among multi-sensitized patients, 236 (28.6%) had co-sensitization to at least one food allergen. The presence of food co-sensitization was significantly associated with a higher number of inhalant sensitizations (*P* < 0.001). Peanut (49.2%), marine fish mix (31.4%), and shrimp (22.0%) were the most prevalent food allergens. The distribution of specific food sensitizations across different numbers of inhalant sensitizations is detailed in Table 6.

**Table 6:** Distribution of food allergens in relation to the number of inhalant sensitizations among multi-sensitized patients with food co-sensitization [*n* (%)].

Food allergen	Number of sensitization [*n* (%)]									Patients positive for allergen (*n*)	*χ* ^2^	*P* value
	2 (*n* = 44)	3 (*n* = 70)	4 (*n* = 78)	5 (*n* = 66)	6 (*n* = 66)	7 (*n* = 34)	8 (*n* = 70)	9 (*n* = 16)	10 (*n* = 0)			
Egg	4 (9.1)	10 (14.3)	6 (7.7)	4 (6.1)	8 (12.1)	6 (17.6)	10 (14.3)	2 (12.5)	–	50	121.730	< 0.001*
Cow's Milk	–	4 (5.7)	2 (2.6)	8 (12.1)	8 (12.1)	4 (11.8)	6 (8.6)	6 (37.5)	–	38	233.625	< 0.001*
Peanut	8 (18.2)	12 (17.1)	38 (48.7)	22 (33.3)	14 (21.2)	6 (17.6)	16 (22.9)	–	–	116	154.504	< 0.001*
Soybean	2 (4.5)	2 (2.9)	2 (2.6)	2 (3.0)	–	–	–	–	–	8	3.847	0.921
Beef	2 (4.5)	4 (5.7)	–	4 (6.1)	4 (6.1)	4 (11.8)	2 (2.9)	–	–	20	43.079	< 0.001*
Mutton	2 (4.5)	4 (5.7)	2 (2.6)	2 (3.0)	4 (6.1)	2 (5.9)	10 (14.3)	2 (12.5)	–	28	188.908	< 0.001*
Marine Fish Mix	12 (27.3)	16 (22.9)	10 (12.8)	6 (9.1)	14 (21.2)	2 (5.9)	8 (11.4)	6 (37.5)	–	74	140.406	< 0.001*
Shrimp	6 (13.6)	4 (5.7)	4 (5.1)	10 (15.2)	10 (15.2)	8 (23.5)	10 (14.3)	–	–	52	132.861	< 0.001*
Crab	8 (18.2)	12 (17.1)	4 (5.1)	8 (12.1)	4 (6.1)	2 (5.9)	8 (11.4)	–	–	46	62.263	< 0.001*
Fruit Mix	–	2 (2.9)	4 (5.1)	–	–	–	–	–	–	6	13.148	0.156
Nut Mix	–	–	6 (7.7)	–	–	–	–	–	–	6	30.905	< 0.001*
Co-sensitization to Food Allergens												
Yes	30 (68.2)	44 (62.9)	60 (76.9)	34 (51.5)	30 (45.5)	12 (35.3)	20 (28.6)	6 (37.5)	–	236	107.622	< 0.001*
No	198	156	76	96	38	20	4	–	2	590		

Data are presented as *n* (column %). Percentages for specific food allergens (rows 1–11) represent the proportion of patients sensitized to that food among those with the specified number of inhalant sensitizations (column). The “Food Allergy” row percentage indicates the prevalence of any food co-sensitization within each inhalant sensitization group. The “Patients Positive for Allergen” column sums patients across all sensitization groups for each allergen.

– indicates zero cases.

Group comparisons were performed using the chi-square (*χ*^2^) test. A *P* value of < 0.05 was considered statistically significant. Percentages may not sum to 100% due to rounding.

* *P* < 0.001.

### 3.6 Distribution of the number of sensitization in different time groups of visits

The distribution of sensitization numbers varied significantly by season of consultation and the period relative to the COVID-19 pandemic (both *P* < 0.001, Table 7). Summer (June–August) accounted for the vast majority of cases across all sensitization number groups. All patients with a high degree of polysensitization (≥7 positive allergens) were diagnosed in the summer season. Following the onset of the pandemic, a marked decrease in the overall number of multi-sensitized cases was observed, and no cases with ≥7 sensitizations were recorded in the post-pandemic period.

**Table 7:** Distribution of the number of sensitizations by season of consultation and COVID-19 pandemic period in multi-sensitized patients [*n* (%)].

Time	Number of sensitization [*n* (%)]									Row total (*n*)	*χ* ^2^	*p* value
	2 (*n* = 228)	3 (*n* = 200)	4 (*n* = 136)	5 (*n* = 130)	6 (*n* = 68)	7 (*n* = 32)	8 (*n* = 24)	9 (*n* = 6)	10 (*n* = 2)			
Season of Consultation											58.475	< 0.001*
Spring (Mar–May)	8 (57.1)	4 (28.6)	2 (14.3)	–	–	–	–	–	–	14		
Summer (Jun–Aug)	190 (26.3)	172 (23.8)	114 (15.8)	118 (16.3)	64 (8.9)	32 (4.4)	24 (3.3)	6 (0.8)	2 (0.3)	722		
Autumn (Sep–Nov)	26 (38.2)	18 (26.5)	14 (20.6)	8 (11.8)	2 (2.9)	–	–	–	–	68		
Winter (Dec–Feb)	4 (18.2)	6 (27.3)	6 (27.3)	4 (18.2)	2 (9.1)	–	–	–	–	22		
Pandemic Period											85.513	< 0.001*
Pre-pandemic (2018–2019)	180 (26.2)	162 (23.5)	104 (15.1)	114 (16.6)	64 (9.3)	32 (4.7)	24 (3.5)	6 (0.9)	2 (0.3)	688		
Post-pandemic (2020–2021)	48 (34.8)	38 (27.5)	32 (23.2)	16 (11.6)	4 (2.9)	–	–	–	–	138		
Overall	228 (27.6)	200 (24.2)	136 (16.5)	130 (15.7)	68 (8.2)	32 (3.9)	24 (2.9)	6 (0.7)	2 (0.2)	826		

Data are presented as *n* (row %). Percentages represent the distribution of sensitization numbers within each seasonal or pandemic period subgroup and sum to 100% across each row.

– indicates zero cases.

Group comparisons were performed using the chi-square (*χ*^2^) test. A *P* value of < 0.05 was considered statistically significant. Percentages may not sum to 100% due to rounding.

* *P* < 0.001.

### 3.7 Distribution of the number of allergens in different ethnic groups

Ethnicity data were available for 688 multi-sensitized patients (Han: 503; Hui: 177; Manchu: 8). A significant difference in the distribution of sensitization numbers was observed among ethnic groups (*P* < 0.001). The Han ethnic group comprised the largest proportion across most sensitization number categories. Detailed data are presented in Table 8.

**Table 8:** Distribution of the number of sensitizations by ethnicity in multi-sensitized patients [*n* (%)].

Ethnic	Number of sensitization/*n* (%)									Row Total (*n*)	*χ* ^2^	*P* value
	2	3	4	5	6	7	8	9	10			
Han	125 (24.9)	132 (26.2)	68 (13.5)	88 (17.5)	42 (8.4)	26 (5.2)	18 (3.6)	2 (0.4)	2 (0.4)	503	53.909	< 0.001*
Hui	53 (29.9)	28 (5.6)	34 (6.8)	26 (5.2)	20 (4.0)	6 (1.2)	6 (1.2)	4 (0.8)	–	177		
Man	2 (25.0)	2 (0.4)	2 (0.4)	–	2 (0.4)	–	–	–	–	8		
Overall	180 (26.2)	162 (32.2)	104 (20.7)	114 (22.7)	64 (12.7)	32 (6.4)	24 (4.8)	6 (1.2)	2 (0.4)	688		

Categorical data are presented as number (*n*) and percentage (%). Group comparisons were performed using the chi-square (*χ*^2^) test. A *P* value of < 0.05 was considered statistically significant.

The dash (–) indicates no cases in that category. Percentages may not sum to 100% due to rounding.

* *P* < 0.001.

## 4 Discussion

Patients with allergic rhinitis (AR) who are multi-sensitized (i.e., allergic to two or more allergens) represent a clinically significant subgroup, often experiencing more severe symptoms and a greater impairment in quality of life compared to mono-sensitized individuals (13–15). The distribution of allergens driving multi-sensitization is known to vary considerably across geographic regions due to environmental and climatic factors (8). Focusing on Ningxia, China, our study delineates the specific profile of multi-sensitized AR patients in this region. We identified pollen allergens, most notably mugwort, followed by ragweed and tree mix, as the predominant triggers. This pattern aligns with the local temperate continental arid climate, which favors the propagation and airborne dispersion of these plants (9).

In addition, it has been observed that patients with multiple allergies combined with allergic rhinitis are more likely to develop asthma than patients with allergic rhinitis with a single allergy (6). These problems make the multi-sensitization of allergic rhinitis a disease problem worthy of attention and treatment.

While multi-sensitization is indeed more common than mono-sensitization (16), its prevalence shows geographical variation. In our Ningxia cohort, 74.4% of sensitized AR patients were multi-sensitized, a figure lower than the >90% prevalence reported in a prior national multi-center study (17), likely reflecting regional differences in allergen exposure profiles. The occurrence of multi-sensitization is influenced by factors such as age, season of onset, co-sensitization to food, and ethnicity.

The closer the species are in classification, the more likely the cross-reaction is to occur. Cross-sensitization arises from IgE recognition of similar protein structures across different allergens, often within botanically related species. In contrast, co-sensitization involves independent IgE responses to allergens that do not share structural homology, possibly due to concurrent environmental exposure (18). It remains debated whether the observed polysensitization pattern results primarily from cross-reactivity, concurrent environmental exposure to multiple allergens, or an individual's inherent atopic predisposition (19).

### 4.1 Differences in the number and type of sensitization

In our cohort, outdoor pollen allergens were the primary causative agents for multi-sensitized AR. Among these, mugwort was the predominant sensitizer, exhibiting the highest proportion, positive rate, and mean sensitization intensity, highlighting its unique role in this region. This pattern indicates a high sensitivity to pollen, with disease manifestations typically being local and seasonal. This can be attributed to several regional factors: (1) The temperate continental arid/semi-arid climate of Ningxia, characterized by low rainfall, high evaporation, and sandy winds, is conducive to the growth and dispersal of mugwort and other pollen-producing plants; (2) Climate change influences pollen season dynamics, plant distribution, and allergen content (20); (3) Air pollutants from human activities can damage pollen grains, potentially increasing environmental allergen concentrations (21). Consequently, for disease management in this population, strategies should combine daily protective measures to minimize exposure with regular monitoring of local airborne pollen levels to facilitate timely AR diagnosis and treatment, which is crucial for asthma prevention.

### 4.2 Differences in the number and intensity of sensitization

The total allergen intensity score may reflect aggregate sIgE concentration in AR patients at a descriptive level, although this metric is not clinically validated. Our results demonstrated a positive correlation between the number of sensitizations and both the total intensity score and the inferred *in vivo* sIgE concentration. Given the established link between elevated total sIgE and allergic diseases, particularly bronchial asthma (22), this heightened immunologic burden might partially explain the increased asthma risk observed in multi-sensitized AR patients (6). However, these findings should be interpreted cautiously, and the proposed indices require further validation in future studies. It is noteworthy, however, that the actual number of patients possessing a high number of sensitizations (e.g., ≥7) was relatively small. To more precisely characterize the relationship between sensitization number and sIgE concentration, future studies with larger sample sizes are warranted.

### 4.3 Sex and age differences in multi-sensitization

We found no significant sex differences in the proportion of multi-sensitization versus mono-sensitization (*χ*2 = 0.295, *P* = 0.587). Consistent with other reports (23, 24), our data confirmed that the number of sensitizations tends to increase with age in both sexes. The observation that boys may acquire a higher number of sensitization earlier in life could partially explain the reported higher proportion of asthma in young boys (25). However, a distinct pattern emerged within the multi-sensitized cohort itself: young women (aged 19−35 years) showed a significant higher rate of multi-sensitization. This contrasts with some studies reporting lower rates of multi-sensitization (particularly to ≥4 allergens) in females across all ages, most notably in early childhood (26). While genetic factors undoubtedly contribute to sex disparities in allergy (27), the underlying mechanisms remain incompletely understood. The age- and sex-specific patterns observed in our study likely arise from a complex interplay of genetic, hormonal, and environmental factors, meriting further investigation.

Although the cross-sectional design of this study prevents causal inference, the observed age-related differences in sensitization profiles are worth noting. Younger children were predominantly mono- or oligo-sensitized, whereas adults exhibited sensitization to a broader range of allergen sources. This pattern is consistent with the hypothesis that cumulative environmental allergen exposure over time may contribute to the progressive broadening of sensitization. However, longitudinal studies are needed to test this hypothesis directly.

### 4.4 The number of allergens and the difference between different allergenic foods

Pollen allergens like mugwort are panallergens. Their conserved protein structures cross pollens and certain plant-derived foods can lead to pollen-pollen and pollen-food cross-reactions (28–30). This cross-reactivity underlies Pollen Food Allergy Syndrome (PFAS), where inhalation of aeroallergens triggers IgE-mediated reactions to structurally similar food proteins (7, 31). PFAS commonly involves allergies to fruits and vegetables (29). Thus, sensitization to one plant pollen allergen can predispose individuals to react to others via cross-reactivity, contributing to the development of multi-sensitization (30, 32). An estimated 30−60% of European food allergy patients have co-existing pollen allergy (7). Supporting this link, one study reported a >60% proportion of food allergy (to items like rice, citrus, and bananas) in children with seasonal AR (19).

### 4.5 Differences in the number of sensitizations and the time of visit

Summer was the peak season for diagnosing multi-sensitized AR. The most frequent allergen combination in these summer cases was mugwort, tree mix, and ragweed, all pollen allergens. Hot, dry summer weather exacerbates nasal symptoms, which may be particularly pronounced in multi-sensitized individuals (33). This seasonal pattern aligns with the pollen season of mugwort locally, which typically spans June to October, peaking in July and August (34). During this period, patients may be in a state of heightened sensitivity, potentially due to cross-reactivity among pollen allergens and other environmental proteins, amplifying allergic responses (19).

The observed decline in multi-sensitization cases after the onset of the COVID-19 pandemic may be associated with public health measures. Reduced outdoor activity (limiting pollen exposure) (35), increased mask usage (providing a physical barrier against pollen) (36), and the altered microclimate behind masks (33) might have collectively contributed to decreased allergen exposure and symptom severity. However, these are speculative hypotheses; our data cannot establish causation, and other unmeasured factors (e.g., changes in healthcare-seeking behavior, reduced access to allergy testing during lockdowns) could also play a role. Future studies are needed to test these hypotheses directly.

### 4.6 Number and nationality of sensitization

Ethnic variations in multi-sensitization could be influenced by genetic background, dietary patterns, or lifestyle factors (29). In our study, no significant ethnic differences in sensitization patterns were detected. This may be due to the limited sample size for certain ethnic groups, the standardized allergen panel used, or shared environmental exposures within the region. The Han ethnicity constituted the majority in our cohort, reflecting both the local demographic composition and potential sampling bias. This highlights a limitation of our retrospective study: the inability to fully account for confounding factors like socioeconomic status, which should be addressed in future research.

### 4.7 Study limitations

This study has several limitations. First, its retrospective, single-center design and the absence of clinical outcome data (e.g., disease severity, asthma comorbidity) preclude assessment of causal relationships or longitudinal changes, limiting generalizability. Second, the fixed allergen panel, while reflecting routine diagnostic practice in this region, did not include grass pollen allergens. The absence of grass pollen data limits the interpretation of the overall sensitization profile, as sensitization to these allergens could not be assessed. However, prior epidemiological data from the Ningxia region indicate that grass pollen sensitization is relatively less common compared to mugwort and ragweed (12). Future studies with broader allergen panels are needed to provide a more complete picture. Third, unmeasured factors such as family history of allergy, rural/urban residence, occupational exposure, and socioeconomic status may influence sensitization patterns and should be addressed in future prospective, multi-center studies. In addition, because the study relied on extract-based testing, we cannot exclude the possibility that cross-reactivity between phylogenetically related allergen sources may have contributed to some of the observed sensitization patterns, potentially leading to apparent oligo-or polysensitization. Future studies using component-resolved diagnostics would be required to distinguish genuine co-sensitization from cross-reactivity.

## 5 Conclusions

In conclusion, multi-sensitized AR patients in Ningxia are predominantly driven by pollen allergens, especially mugwort, and exhibit a distinct profile characterized by a higher sIgE burden, specific demographic patterns, and frequent food co-sensitization, largely explainable by PFAS. Our findings emphasize the importance of comprehensive allergen testing to identify this high-risk subgroup. Management should be informed by local pollen monitoring and remain vigilant for comorbid asthma and food allergy. The observed impact of COVID-19 measures further highlights the effectiveness of targeted exposure reduction strategies.

## Data Availability

The datasets used and/or analysed during the current study are available from the corresponding author on reasonable request.
